# Structure of triosephosphate isomerase from *Cryptosporidium parvum*

**DOI:** 10.1107/S1744309111019178

**Published:** 2011-08-16

**Authors:** Trang N. Nguyen, Jan Abendroth, David J. Leibly, Kristen P. Le, Wenjin Guo, Angela Kelley, Lance Stewart, Peter J. Myler, Wesley C. Van Voorhis

**Affiliations:** a Seattle Structural Genomics Center for Infectious Disease (SSGCID), USA; b Department of Medicine, Division of Allergy and Infectious Diseases, School of Medicine, University of Washington, Box 356423, Seattle, WA 98195-6423, USA; c Emerald BioStructures Inc., 7869 NE Day Road West, Bainbridge Island, WA 98110, USA; d Seattle Biomed, 307 Westlake Avenue North, Suite 500, Seattle, WA 98109, USA

**Keywords:** glycolysis, triosephosphate, triosephosphate isomerases, metabolism, *Cryptosporidium parvum*

## Abstract

*Cryptosporidium parvum* is one of several *Cryptosporidium* spp. that cause the parasitic infection cryptosporidiosis. Cryptosporidiosis is a diarrheal infection that is spread *via* the fecal–oral route and is commonly caused by contaminated drinking water. Triosephosphate isomerase is an enzyme that is ubiquitous to all organisms that perform glycolysis. Triosephosphate isomerase catalyzes the formation of glyceraldehyde 3-phosphate from dihydroxyacetone phosphate, which is a critical step to ensure the maximum ATP production per glucose molecule. In this paper, the 1.55 Å resolution crystal structure of the open-loop form of triosephosphate isomerase from *C. parvum* Iowa II is presented. An unidentified electron density was found in the active site.

## Abstract 2

The crystal structure of the ubiquitous glycolytic enzyme triosephosphate isomerase from *C. parvum* in the open-loop conformation was determined at a resolution of 1.55 Å.

## 1. Introduction

**Figure 1:**
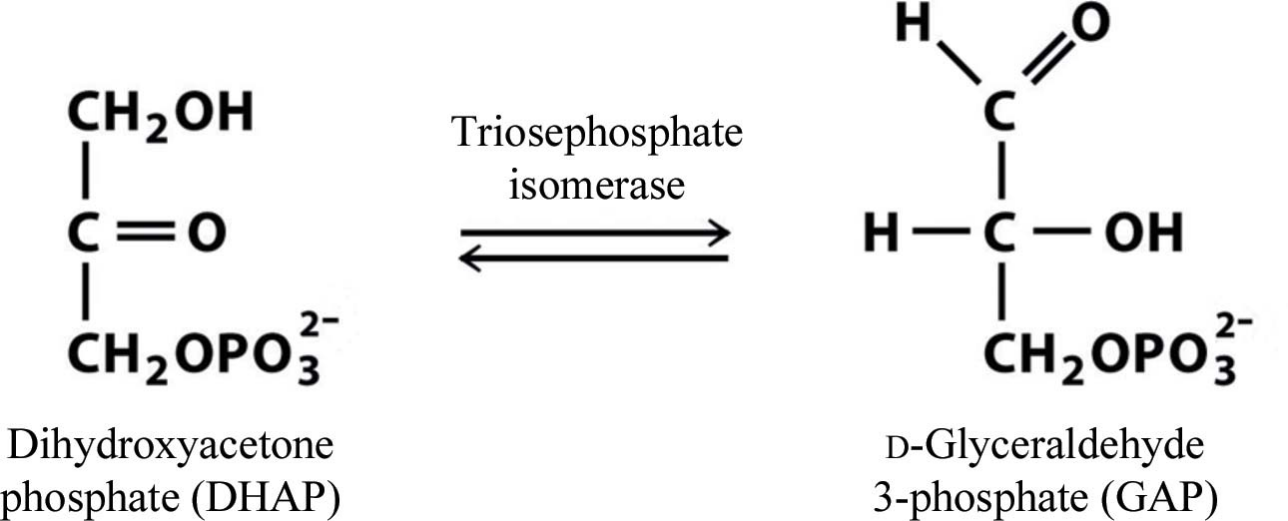
Reaction catalyzed by TIM.

Triosephosphate isomerase (TIM) is an enzyme that is only active in its dimeric form and that has been found in almost all organisms that participate in glycolysis. The aldolase (fourth) step of glycolysis ends with two products: dihydroxyacetone phosphate (DHAP) and d-­glyceraldehyde 3-phosphate (GAP). Only GAP is able to proceed to the next phase of glycolysis. During the fifth glycolysis step, TIM catalyzes the reversible isomerization of dihydroxyacetone phosphate (DHAP) to d-glyceraldehyde 3-phosphate (GAP) (see Fig. 1 ▶). TIM is essential for maximum energy production. Because of its important role in glycolysis, triosephosphate isomerase has been described as an ‘an attractive target for drug design against parasites’ (Mande *et al.*, 1994 ▶).

As of January 2011, 118 structures of TIM have been released in the PDB. The first apo structure of TIM from chicken muscle was deposited in 1976 (PDB entry 1tim; Banner *et al.*, 1976 ▶). This was followed in 1990 by the deposition of the first ligand-bound structure of yeast TIM in the PDB [PDB entries 1ypi (Lolis *et al.*, 1990 ▶) and 2ypi (Lolis & Petsko, 1990 ▶)]. Since then, many other TIM structures from 31 organisms, with and without ligands, have been deposited. TIM structures have been observed in both a closed and an open form. The open form has been observed for apo TIM, while ligand binding induces the closure of a loop. However, one ligand-bound structure of TIM from *Plasmodium falciparum* has been described with both an open and a closed conformation of the loop (Parthasarathy *et al.*, 2003 ▶).

*Cryptosporidium parvum* is a protozoan parasite that causes cryptosporidiosis. Unlike the closely related *C. hominis*, which colonizes humans almost exclusively, *C. parvum* can infect a variety of vertebrates *via* transmission of oocytes. Together, *Cryptosporidium* spp. are a leading cause of morbidity and mortality in mammals, resulting primarily in gastrointestinal problems and diarrhea, or death in the immunocompromised. Currently, there are no reliable treatments for cryptosporidiosis. Paromomycin, azithromycin and nitazoxanide have all shown partial efficacy in reducing disease severity in individuals by shortening diarrheal duration (Gargala, 2008 ▶; Mead, 2002 ▶). However, for immunocompromised patients it is necessary to restore an effective immune response in addition to antimicrobial treatment of the *Cryptosporidium* infection itself (Gargala, 2008 ▶). Here, we present the 1.55 Å crystal structure of the apo form of TIM from *C. parvum* Iowa II as part of a structure-based drug-discovery effort.

## 2. Methods

### 2.1. Protein expression and purification

The gene encoding the full-length (Met1–Gln250) triosephosphate isomerase (UniProt Q5CSE7; residues 1–250) was PCR-amplified in a 96-well format using genomic DNA as the template. The primers were designed with an additional ligase-independent cloning (LIC; Aslanidis & de Jong, 1990 ▶) sequence at their 5′ ends that is complimentary to the LIC sequence in the plasmid vector. Purified PCR products were then cloned *via* LIC into the AVA0421 expression vector (Quartley *et al.*, 2009 ▶), which provides a C3-cleavable hexahistidine tag at the N-terminus of the expressed protein with the sequence MAHHHHHHMGTLEAQTQ′GPGS. The recombinant plasmids were then transformed into *Escherichia coli* Rosetta Oxford strain [BL21*(DE3)-R3-pRARE2] cells for expression testing. Soluble protein was observed and the target was moved on to large-scale expression. The protein was given the internal designation CrpaA.01119.a.A1 and will be referred to as CrpaA.01119.a.A1/TIM.

Starter cultures of LB broth with appropriate antibiotics were grown for ∼18 h at 310 K. ZYP-5052 auto-induction medium was freshly prepared as per UW-PPG protocols (Studier, 2005 ▶; Choi *et al.*, 2011 ▶). Antibiotics were added to 2 l bottles of sterile auto-induction medium. The bottles were inoculated with the entire overnight culture. Inoculated bottles were then placed into a LEX Bioreactor (Harbinger Biotechnology, Toronto, Ontario). Cultures were grown for ∼24 h at 298 K. The temperature was reduced to 288 K and the culture was grown for a further ∼60 h. To harvest, the medium was centrifuged at 4000*g* for 20 min at 277 K. The cell paste was flash-frozen in liquid nitrogen and stored at 193 K.

The frozen pellet was thawed and completely resuspended in lysis buffer (20 m*M* HEPES pH 7.2–7.4, 300 m*M* NaCl, 5% glycerol, 30 m*M* imidazole, 0.5% CHAPS, 10 m*M* MgCl_2_, 3 m*M* β-mercapto­ethanol, 1.3 mg ml^−1^ protease-inhibitor cocktail and 0.05 mg ml^−1^ lysozyme). The resuspended cell pellet was then disrupted on ice for 15 min with a Branson Digital Sonifier 450D (set to 70% amplitude, with alternating cycles of 5 s pulse-on and 10 s pulse-off). The cell debris was incubated with 20 units ml^−1^ Benzonase nuclease (EMD, San Diego, California, USA) at room temperature for at least 40 min and clarified by centrifugation in a Sorvall RC5 (Thermo Scientific, Pittsburgh, Pennsylvania, USA) at 10 000 rev min^−1^ for 60 min at 277 K. The tagged TIM from *C. parvum* was separated from the clarified cell lysate by immobilized metal-affinity chromatography (IMAC) on a HisTrap FF 5 ml column (GE Biosciences, Piscataway, New Jersey, USA) equilibrated with binding buffer (20 m*M* HEPES pH 7.0, 300 m*M* NaCl, 5% glycerol, 30 m*M* imidazole, 1 m*M* tris(2-­carboxyethyl)phosphine (TCEP). The recombinant protein was eluted in 500 m*M* imidazole plus 1 m*M* TCEP. Cleavage of the N-­terminal His tag was accomplished by overnight incubation with His-MBP-3C protease at 277 K while dialyzing in 25 m*M* HEPES pH 7.0, 300 m*M* NaCl, 5% glycerol, 1 m*M* TCEP, 0.025% sodium azide. The cleaved protein was recovered both in the flowthrough and in the wash fractions, utilizing the same buffers as for the initial IMAC step. After affinity-tag cleavage, a tag remnant GPGS was left at the N-terminus of the full-length protein. The protein was further resolved by size-exclusion gel chromatography (SEC; Superdex 75 26/60; GE Bio­sciences, Piscataway, New Jersey, USA). Pure fractions collected in SEC buffer (20 m*M* HEPES pH 7.0, 300 m*M* NaCl, 2 m*M* DTT, 5% glycerol) as a single peak were analyzed using SDS–PAGE and SimplyBlue Safestain (Invitrogen, Carlsbad, California, USA). The protein was then pooled, concentrated to 75.3 mg ml^−1^, flash-frozen and stored at 193 K in SEC buffer. Further details can be found in Bryan *et al.* (2011 ▶).

### 2.2. Crystallization

Thawed protein was used to set up four sparse-matrix screens (JCSG+, Cryo and Wizard from Emerald BioStructures, Bainbridge Island, Washington, USA and PACT from Molecular Dimensions, Apopka, Florida, USA) following an extended Newman strategy (Newman *et al.*, 2005 ▶). 0.4 µl protein solution was mixed with 0.4 µl well solution and equilibrated against a 100 µl reservoir using 96-well Compact Jr crystallization plates (Emerald BioStructures). Crystals suitable for diffraction studies were found in condition G8 from the PACT screen: 100 m*M* Bis-Tris propane pH 7.5, 200 m*M* sodium sulfate, 20% PEG 3350. The crystals were cryoprotected with an additional 25% ethylene glycol.

### 2.3. Data collection and structure determination

An X-ray diffraction data set was collected on 25 October 2009 on ALS beamline 5.0.3 at the Berkeley Center for Structural Biology, which is equipped with a 3 × 3 tiled ADSC Q315r detector. 180 images were collected at a wavelength of 0.9774 Å with a crystal-to-­detector distance of 225 mm, a ϕ-slicing of 1° per image and an exposure time of 1 s. The diffraction data were processed with *XDS*/*XSCALE* (Kabsch, 2010 ▶; see Tables 1 ▶ and 2 ▶). The crystals belonged to space group *P*2_1_; the diffraction limit was 1.55 Å.

**Figure 2:**
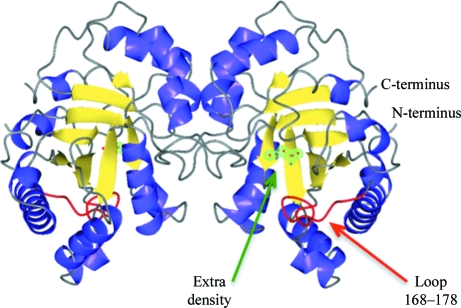
CrpaA.01119.a.A1/TIM dimer. View of the dimer of CrpaA.01119.a.A1/TIM, which has the typical TIM-barrel fold. The active-site loop (Pro168–Ala178) is highlighted in red and is in the typical ‘open’ conformation. Despite the open conformation of the loop, extra *F*_o_ − *F*_c_ electron density can be observed in proximity to the active site. The structure was deposited with UNK atoms as indicators for unidentified atoms (highlighted with red spheres).

Calculation of the packing density (Matthews, 1968 ▶) suggested two molecules of CrpaA.01119.a.A1/TIM per asymmetric unit, with a *V*_M_ of 2.56 Å^3^ Da^−1^ and 52% solvent content. A search of the PDB for sequence homology yielded a TIM from *P. falciparum* (PDB entry 2vfd; Gayathri *et al.*, 2009 ▶) as the closest homolog, with 47% sequence identity. The search model was derived from monomer *A* of PDB entry 2vfd with the *CCP*4 program *CHAINSAW* (Stein, 2008 ▶; Winn *et al.*, 2011 ▶) by trimming nonconserved amino acids to the C^γ^ atom while maintaining conserved residues (Schwarzenbacher *et al.*, 2004 ▶). Molecular replacement was performed with the *CCP*4 program *Phaser* (McCoy *et al.*, 2007 ▶) using data between 20 and 3.5 Å resolution. The two molecules could be placed with high scores: RFZ1 = 13.4, TFZ1 = 11.8, LLG1 = 207, RFZ2 = 11.3, TFZ2 = 23.6, LLG = 797. The model was then extended by *ARP*/*wARP* (Langer *et al.*, 2008 ▶), which built 471 residues in six chains with *R*_work_ = 0.208 and *R*_free_ = 0.273. The model was then iteratively extended manually using *Coot* (Emsley *et al.*, 2010 ▶) followed by cycles of reciprocal-space refinement with the *CCP*4 program *REFMAC*5 (Murshudov *et al.*, 2011 ▶). The final model could be refined with one TLS group per chain to an *R*_work_ of 0.157 and an *R*_free_ of 0.188 with good stereochemistry (see Fig. 2 ▶). The model was validated with the validation tools in *Coot* and *MolProbity* (Chen *et al.*, 2010 ▶). The final model extended from Ser2 to Gln250 in both chains. For each chain, a sodium ion was modelled binding to the carbonyl C atoms of Cys225 and Leu228 and to the hydroxyl group of Tyr5. In both σ_A_-weighted *F*_o_ − *F*_c_ maps contoured at 3σ and σ_A_-­weighted 2*F*_o_ − *F*_c_ maps contoured at 1σ a blob of extra density could be observed in both chains in the proximity of Gly234. As neither of the components of the crystallization drop could serve as a good interpretation, this density was modelled as unidentified (UNK; see §[Sec sec3]3).

## 3. Results and discussion

SSGCID target CrpaA.01119.a.A1/TIM crystallized in standard sparse-matrix screens. A high-resolution data set was collected from the original screens without further optimization.

CrpaA.01119.a.A1/TIM crystallized with two monomers in the asymmetric unit. Interface analysis using *PISA* (Krissinel & Henrick, 2007 ▶) confirmed that this was the native dimer. The dimer buries a surface of 1600 Å^2^ per monomer compared with a surface area of ∼11 000 Å^2^ per monomer; the free binding energy Δ*G* ^int^ was estimated as −96 kJ mol^−1^. Dimers are the active quaternary structure of TIMs.

The fold of CrpaA.01119.a.A1/TIM is the typical TIM-barrel fold consisting of a barrel of eight β-strands surrounded by helices (see Fig. 2 ▶). The two monomers are very similar: 249 C^α^ atoms can be superimposed with an r.m.s.d. of 0.2 Å. An *SSM* (Krissinel & Henrick, 2004 ▶) search of the PDB reveals several other TIMs with r.m.s.d.s of around 1 Å.

Early studies on TIM revealed that the protein can exist in an open form and a closed form (Wierenga *et al.*, 1991 ▶). The closure of a loop, commonly called ‘loop 6’ or the ‘active-site loop’, is typically observed in ligand-bound structures. The closed loop is thought to protect the reaction intermediates from the environment. The open conformation is typically found in apo structures. However, in *P. falciparum* TIM both open and closed conformations can be observed in a high-resolution (1.1 Å) ligand-bound structure (Parthasarathy *et al.*, 2003 ▶).

**Figure 3:**
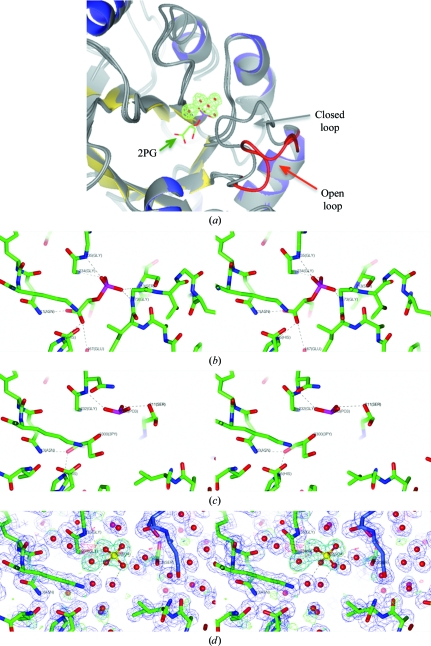
Extra density in the active site of CrpaA.01119.a.A1/TIM. (*a*) A close-up of the active-site loop (red) of CrpaA.01119.a.A1/TIM and the extra blob of density shown in the same colours as in Fig. 2 ▶. The structure of TIM from *T. brucei* in complex with 2-phosphoglycerate (2PG) is superimposed (PDB entry 4tim) and shown in light-grey ribbons, with the cofactors shown as colored sticks. The active-site loop of CrpaA.01119.a.A1/TIM is in the open conformation, while the active-site loop of ligand-bound TIM from *T. brucei* is closed. The extra density in the CrpaA.01119.a.A1/TIM structure, shown as σ_A_-weighted *F*_o_ − *F*_c_ density contoured at 3σ, almost superimposes with the phosphate group of 2PG in *T. brucei* TIM, but extends in a different direction. (*b*) A stereo figure of the 2PG-binding environment in *T. brucei* TIM (PDB entry 4tim) shown in the same orientation as in (*a*). 2PG is bound to the protein by a tight hydrogen-bond network. Residues to the right of the ligand are part of the closed active-site loop, which is in the closed conformation. (*c*) A stereo figure of the ligand-binding environment in *P. falciparum* TIM (PDB entry 1o5x) shown in the same orientation as in (*b*). The 2PG ligand is cleaved, possibly as a consequence of radiation damage, to 2-oxoglycerate (3PY) and metaphosphate (PO3). The active-site loop is in the open conformation. (*d*) A stereo figure of the environment of the unidentified electron density in CrpaA.01119.a.A1 together with the σ_A_-weighted OMIT 2*F*_o_ − *F*_c_ electron density at 1σ in blue and the σ_A_ OMIT *F*_o_ − *F*_c_ electron density at ±3σ in green/red. One protein molecule is shown with green C atoms and the crystallographic symmetry mate is shown with purple C atoms. The unidentified electron-density blob is in proximity to the phosphate group of 2PG in *T. brucei* TIM; however, the glycerate group of 2PG does not match the density. While the hydrogen-bonding partners of 2PG to the left in (*b*) are conserved compared with CrpaA.01119.a.A1/TIM, the different conformations of the active-site loop render the binding environment to the right significantly different. The unidentified electron-density blob also superimposes with the metaphosphate in *P. falciparum* TIM. The right part of the extra electron density has a somewhat tetrahedral shape. However, a tentatively modelled sulfate ion (ball-and-stick model) together with a water molecule make only very few interactions with the protein. Refinement indicates that sulfate would only be at low occupancy.

In CrpaA.01119.a.A1/TIM the active-site loop extends from residues Pro168 to Ala178 and is in the open conformation (Fig. 2 ▶). While the ‘open’ conformation is typical of apo structures, a blob of extra electron density is observed in proximity to the active site of the CrpaA.01119.a.A1/TIM structure (see Figs. 2 ▶ and 3 ▶). This blob of density almost superimposes with the phosphate group of 2-phospho-glycerate (2PG) in the TIM structure from *Trypanosoma brucei* (PDB entry 4tim; Noble *et al.*, 1991 ▶; see Fig. 3 ▶ *a*). The two structures superimpose with an r.m.s.d. of 1.1 Å over 239 residues. Figs. 3 ▶(*b*) and 3 ▶(*d*) compare the 2PG-binding environment in *T. brucei* TIM with the extra blob of density in the CrpaA.01119.a.A1/TIM structure. In the *T. brucei* TIM structure 2PG is bound to the protein with a tight hydrogen-bond network. These interactions involve Asn11 N^δ2,^ His95 N^∊2^, Glu167 O^∊1^, Leu232 N (not shown in Fig. 3 ▶ *b*), Gly234 N and Gly235 N, which are structurally conserved between the two structures. However, the extra density does not entirely match the phosphate group. Further interactions between 2PG and *T. brucei* TIM involve residues from the active-site loop: Gly173 N and Ser213 N. As the active-site loop in the CrpaA.01119.a.A1/TIM is in the open conformation, the latter two residues are not structurally conserved between the two structures.

In the structure of *P. falciparum* TIM (PDB entry 1o5x; Parthasarathy *et al.*, 2003 ▶) the active-site loop of subunit *B* is mostly in an open conformation, even though ligands are observed in the active site. In this structure, 2PG has been broken down to into two fragments, which were tentatively identified as 2-oxoglycerate and metaphosphate (see Fig. 3 ▶ *c*). The 2-oxoglyerate group makes similar interactions with the protein as described for the *T. brucei* structure above. The metaphosphate occupies a similar location as the un­identified density in the CrpaA.01119.a.A1/TIM structure; however, it does not fill it entirely.

Fig. 3 ▶(*d*) shows a close-up of the active-site region of CrpaA.01119.a.A1/TIM and the unidentified electron density. Owing to similarity to the *T. brucei* and *P. falciparum* enzymes, a phosphate group appears to be a likely explanation for the right part of the extra electron density. With an additional water molecule on the left, the density could be fully explained. While the crystallization drop did not contain phosphate, it did contain the isosteric sulfate ion at a concentration of 200 m*M*. In Fig. 3 ▶(*d*), we have tentatively modelled a sulfate ion and an additional water molecule into the unidentified density. The somewhat tetrahedral shape of the right part of the density would support this. The sulfate group would make one direct interaction with protein (Gly235 N) and several interactions with water molecules. However, refinement indicates that the occupancy of this sulfate would be low.

As the identity of the ligand could not be fully established, we deposited the structure with UNK atoms in the active site. Further crystallization experiments may identify the ligand and might establish the relationship between the conformation of the active-site loop and ligand binding in CrpaA.01119.a.A1/TIM.

## 4. Conclusions

This paper describes the purification, crystallization and structure solution of *C. parvum* triosephosphate isomerase (CrpaA.01119.a.A1/TIM). The presented 1.55 Å resolution structure shows the typical TIM fold and contains a dimer, which is consistent with previously observed structures. The active-site loop is in the open conformation which is typical for apo structures. An unidentified blob of electron density occupies the location of phosphate moieties of ligands in other TIM structures. However, phosphate or sulfate from the crystallization buffer can only partially explain the extra density. Additional studies would need to be performed in order to identify this ligand. Furthermore, ligand-binding studies could establish the relation between compound binding and the conformation of the active-site loop.

## Supplementary Material

PDB reference: triosephosphate isomerase, 3krs

## Figures and Tables

**Table 1:** Data-collection statistics Values in parentheses are for the highest of 20 resolution shells.

Beamline	ALS 5.0.3
Wavelength (Å)	0.9765
Space group	*P*2_1_
Unit-cell parameters (Å, °)	*a* = 53.56, *b* = 71.93, *c* = 75.38, β = 106.4
Resolution range (Å)	20–1.55 (1.59–1.55)
Mean *I*/σ(*I*)	12.9 (2.9)
*R*_merge_†	0.077 (0.364)
Completeness (%)	99.0 (91.3)
Multiplicity	3.6 (2.5)
No. of unique reflections	79305 (5364)
Wilson *B* factor (Å^2^)	10.4

† *R*_merge_ = 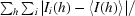 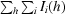.

**Table 2:** Refinement and model statistics Values in parentheses are for the highest of 20 resolution shells.

Beamline	ALS 5.0.3
Resolution range (Å)	20–1.55 (1.59–1.55)
*R*_cryst_†	0.157
*R*_free_†	0.188
R.m.s.d. bonds (Å)	0.014
R.m.s.d. angles (°)	1.42
Protein atoms	3913
Nonprotein atoms	859
Mean *B* factor (Å^2^)	9.9
Residues in favored region	456 [97%]
Residues in allowed region	6 [1.3%]
Residues in disallowed region	6 [1.3%]
*MolProbity*‡ score (percentile)	1.46 [90th]
PDB code	3krs

† *R*_cryst_ = 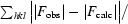 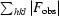. The free *R* factor was calculated using an equivalent equation with the 5% of the reflections that were omitted from the refinement.

‡ Chen *et al.* (2010 ▶).
